# Transvenous Closure of Patent Foramen Ovale: Preliminary Results with a New Self-Expanding Nitinol Wire Mesh in a Swine Model

**DOI:** 10.4061/2009/943453

**Published:** 2009-09-09

**Authors:** F. Krizanic, M. Sigler, H. R. Figulla

**Affiliations:** ^1^Department of Cardiology, Clinic of Internal Medicine I, Friedrich Schiller University, 07740 Jena, Germany; ^2^Pediatric Cardiology and Intensive Care Medicine, Georg August University, D 37099 Goettingen, Germany

## Abstract

*Objectives*. The transvascular closure of patent foramen ovale (PFO) with self-expanding devices carries the risk of left atrial thrombus formation related to material protruding into the left atrium. Thus, we developed a novel device with flat left atrial disc geometry. We evaluated feasibility, handling, and biocompatibility in a porcine animal model. *Methods*. Implantation of an Occlutech Figulla PFO device was performed in 10 mini pigs using fluoroscopy and intra-cardiac ultrasound after transseptal puncture of the interatrial septum. Angiographic follow-up was performed after six and twelve weeks. *Results*. Implantation was successful in 100%. There were no further implant related complications. One procedure related death occurred, as one animal died of ventricular tachycardia due to mispunture of the interatrial septum. Angiographic studies showed no residual shunt during follow-up. Histopathological evaluation could demonstrate partial neoendothelialization after 6 weeks with completion after 12 weeks. The devices were incorporated into connective tissue containing fibro muscular cells. An only mild inflammatory reaction was detected locally related to the polyester fibers. *Conclusion*. In terms of feasibility and handling, the new device does not seem to be inferior to other presently used implantation systems. Good biocompatibility was demonstrated with rapid and complete neoendothelialization.

## 1. Introduction

 The patent foramen ovale is an open passageway between the superior limb of the septum secundum of the right atrial side and the septum primum of the left atrial side. It represents the physiological interatrial communication throughout fetal life.

 Functional closure occurs immediately after birth which is usually followed by anatomical closure within the first weeks postnatally. In about 27% of humans, this anatomical fusion does not occur [1]. In case of a right-to-left shunt, paradoxical embolism may occur with the result of transient ischemic attack or stroke. In 35–44% of patients with ischemic stroke the origin remains unknown (cryptogenic) [2]. Several studies have shown that the prevalence of a PFO in these patients is significantly higher than in control groups. Whereas a PFO is found in 44–66% of patients with cryptogenic stroke, in patients with known causes of stroke it is found in only 9–27%. There is a high recurrence rate of a second thromboembolic event despite medical treatment ranging between 2% and 15% per year, especially in the age group >55 years. With this high prevalence, there is a need for preventative measures in the elderly [3].

 Transcatheter interventional closure of a patent foramen ovale has become a routine procedure in adults with a low risk of periprocedural complications and good long-term results in a large retrospective study [4–7]. Since the first implantation of an occlusion device into an intra-atrial communication in 1976 [8], different device systems have been designed to improve feasibility, efficacy, and safety.

## 2. Methods

### 2.1. Occlutech Figulla PFO Device

**Figure 1:**
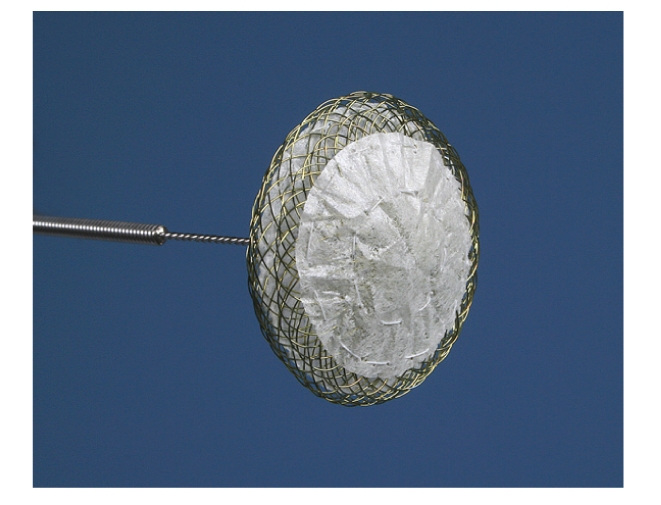
View of the Occlutech Figulla PFO device (23/25 mm) prior to implantation.

**Figure 2:**
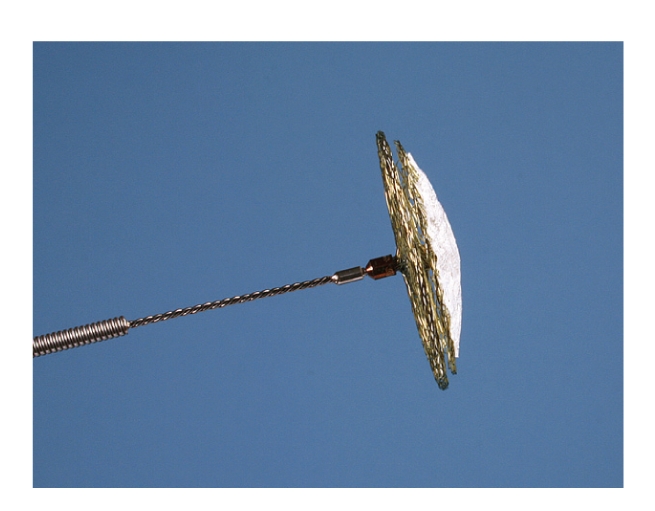
View of the Occlutech Figulla PFO device (23/25 mm) prior to implantation.

The device consists of a nitinol wire mesh (0.12 mm) forming two flexible retention discs (diameter 23/25 mm) using a braided unique technology which allows forming a single hub on the proximal side (Figures 1 and 2). The material is characterized by a shape memory effect which allows self-expansion. The two discs are linked by a 3 mm central waist. Two PET patches (polyethylenterephtalat) assure complete closure after implantation.

 The novelty is that the left atrial disc only consists of a single layer wire mesh without having a hub which therefore minimizes the amount of material on the left atrial side.

### 2.2. Animal Model

The aim of present study was the implantation of a PFO device in Goettingen mini pigs, which are frequently used as animal model for the experiments closely related to human medicine. Artificial creation of PFO in piglets is feasible by percutaneous atrial septal puncture. PFO prevalence in mini pig is identical to humans, and the microscopic structures are very similar to humans [9]. The mini pig PFO model appears useful to evaluate new interventional closure technologies due to comparability in microscopic features. The implantation of a device in an naturally existing atrial septal defect would be a more realistic test than artificial creation, but therefore a larger number of animals in consideration of PFO prevalence would have been needed.

 Preprocedural anaesthesia was achieved by sedation with 10 mg/kg ketamine and 0.4 mg/kg xylazin; the animals underwent endotracheal intubation using propofol and were mechanically ventilated with 1-2% isoflurane and 30% N_2_O. Electrocardiogram, invasive blood pressure measurement, heart rate, oxygen saturation, tidal volume, and end-tidal CO_2_ were monitored. Implantation was performed in 10 mini pigs (mean weight 36 kg, mean age 18 months) under guidance of fluoroscopy and by intracardiac echocardiography (ICE). After transseptal puncture of the interatrial septum with an transseptal needle inside a catheter (Brockenbrough guidance sheath with transseptal punture set (Brockenbrough Needle, Medtronic, Inc. Mounds View, Minnesota) using a nine-French delivery system sheath, the artificial defect was passed with a 150 cm long guidewire. It was inserted into the left atrium till reaching the upper left pulmonary vein after giving of heparin 200 U/kg.

 The device was loaded and advanced via the guiding sheath under fluoroscopic control. After deployment of the left atrial disc the system was pulled back and was finished by deployment of the proximal disc on the right atrial side. A relevant residual shunt was excluded by injection of contrast media before disconnection of the occluder.

Clopidogrel 75 mg (3 months) and acetylsalicylacid 100 mg (6 months) per day were administered after implantation.

 All mini pigs received human care in compliance with the “guide for the care and use of laboratory animals” published by the US National Institutes of Health (NIH Publication No. 85–23, revised 1996). The study was approved by the local governmental animal ethics committee.

### 2.3. Follow-up Examination

Angiographic follow-up examinations were performed in one animal after six weeks and in eight animals after twelve weeks. After the angiography, the animals were killed by intravenous administration of potassium chloride.

### 2.4. Tissue Preparation

The tissue block containing the implant was fixed in formalin (buffered 4%) for histological examination and embedded in the hard resin methylmethacrylat (Technovit 9100; Kulzer & Co, Wehrheim, Germany). After hardening of the resin, the specimen was subsequently sectioned in slices of 0.8 mm using a diamond cutter. These slices were ground down to 10–30 *μ*m by a rotational grinder as previously described [10]. Staining of the specimen was performed with Richardson blue.

## 3. Results

Implantation was successful in all animals (100%; *n* = 10/10). The mean time from intubation to device implantation was 138.9 ± 42 minutes. The procedure time varied considerably due to the different anatomical features of the porcine and the human intra-atrial septum.

 Two of the 10 animals died ahead of scheduled end of the experiments. One mini pig died in cardiogenic shock due to ventricular tachycardia which was caused by recurrent mispuncture of the IAS after completion of the implantation procedure. A second animal died in respiratory failure due to pneumonia after 3 months. Another laboratory animal developed transient pericardial effusion without hemodynamic relevance, which resolved without therapeutic intervention.

 The mean implantation time was 127 ± 46 days. Angiographic studies during followup showed no relevant residual shunt.

### 3.1. Macroscopic Examination

**Figure 3:**
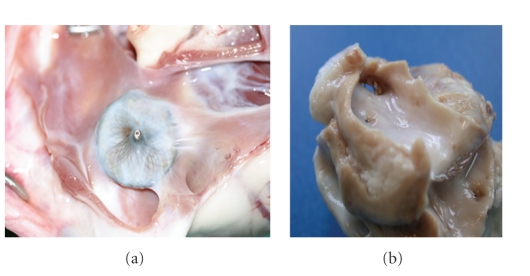
Macroscopic aspect of the right atrial disc (a), and the left atrial disc (b) of an explanted Occlutech Figulla PFO device after an implantation time of 12 weeks. Both discs are completely covered by fibrous tissue without any thrombus deposition.

The devices were covered with a thin shining layer of whithish tissue (Figure 3). On the right atrial side coverage was complete; on the left atrial side small areas were covered incompletely depending on the different anatomical structures in comparison to human heart because of the intra-atrial septum diameter which led to incomplete adaptation. Nevertheless no thrombus formation could be detected on the surface.

 No deformations or wire strut fractures could be seen macroscopically or under fluoroscopic control.

### 3.2. Histology

**Figure 4:**
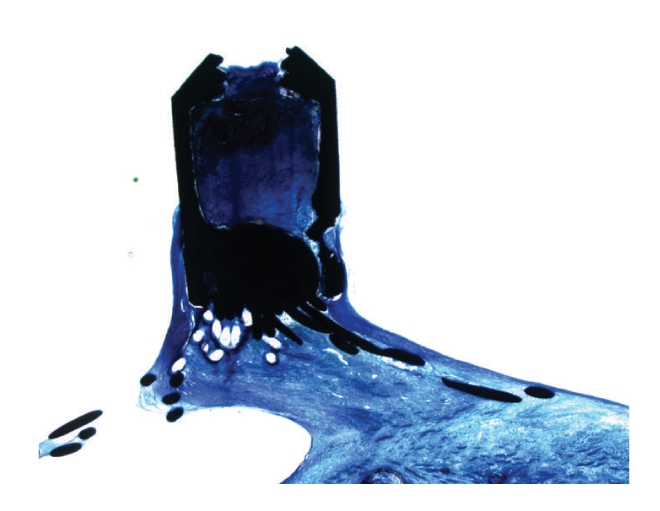
The central pin represents the right atrial hub without signs of corrosion.

**Figure 5:**
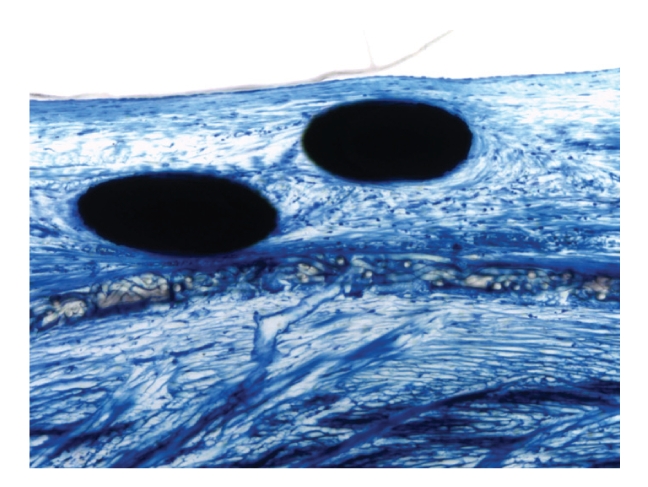
Micrograph of metal struts of the occluder (black) on the right atrial side. The struts are completely covered by fibrous tissue and endothelium. Magnification 100×, Richardson blue staining.

Metal struts of the nitinol wire mesh and the right atrial central hub were smooth without signs of corrosion (Figure 4). The implant was incorporated in dense connective tissue containing fibromuscular cells, capillaries, and small vessels (Figure 5).

**Figure 6:**
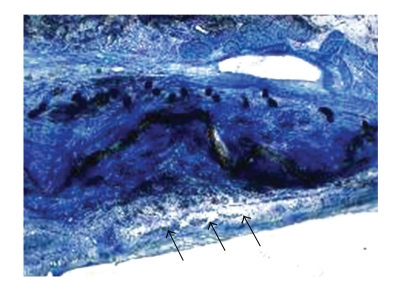
The PET-patch on the left atrial side (arrows) is completely covered by fibrous tissue and endothelium without major inflammatory reactions. Magnification 16×, Richardson blue staining. PET: polyethylenterephtalat.

 Some multinucleated foreign body giant cells were found neighbouring the polyester fibres (Figure 6). Circumscribed mild lymphocytic infiltration was observed with a loose distribution within the implant, but constantly locally related to the polyester fibres.

## 4. Discussion

It is appreciated that a PFO is a frequent cause of cerebral embolism. Until today there is a continuous debate as to how these patients should be treated [11–13]. Multiple data point to higher prevalence of PFO in patients with a second cryptogenic stroke and in patients older than 55 years and those with septal aneurysm [14–16]. An additional risk of stroke recurrence is presented by a large PFO defect diameter [17, 18].

 Many different PFO occlusion devices are marketed currently with remarkable differences in device design. Protrusion of implant material on the left atrial side could be identified as a risk factor for thrombus formation or even thromboembolism after interventional therapy of a PFO [19–22]. Thus we developed a new PFO occlusion device made from a nitinol meshwork using a unique patented braiding technology with reduces left atrial foreign material.

It is regarded mandatory to examine biocompatibility of human implants prior to introduction into clinical use [23]. In the present study, the device was implanted in 10 Goettingen mini pigs. Macroscopic, angiographic, and histopathological followup did not show any superficial thrombus formation, wire fractures, or major inflammatory reactions.

 Nevertheless, few lymphocytic infiltrations and some foreign giant body cells neighbouring the polyester fibres were seen in the implant. The extent of inflammatory reactions was similar to those described for other devices [24, 25]. Overall, good biocompatibility of this novel PFO occluder could be demonstrated in our study. Long-term observations have to answer the question if the improved geometry of the left atrial disc can reduce thromboembolic complications following interventional PFO closure.

 Perfecting device technology together with advanced identification of patients who mostly benefit from interventional therapy will further improve the outcome of patients with PFO.

## 5. Conclusion

The Occlutech Figulla PFO device appears to be safe and feasible for percutaneous PFO closure. Furthermore, the data presented demonstrate good biocompatibility of the device and therefore complement the first positive clinical data [26].
